# Network Distance-Based Simulated Annealing and Fuzzy Clustering for Sensor Placement Ensuring Observability and Minimal Relative Degree

**DOI:** 10.3390/s18093096

**Published:** 2018-09-14

**Authors:** Daniel Leitold, Agnes Vathy-Fogarassy, Janos Abonyi

**Affiliations:** 1 Department of Computer Science and Systems Technology, University of Pannonia, Egyetem u. 10, H-8200 Veszprém, Hungary; leitold@dcs.uni-pannon.hu (D.L.); vathy@dcs.uni-pannon.hu (A.V.-F.); 2 MTA-PE Lendület Complex Systems Monitoring Research Group, University of Pannonia, Egyetem u. 10., POB. 158, H-8200 Veszprém, Hungary

**Keywords:** sensor placement, network science, fuzzy clustering, simulated annealing, structural observability, relative degree, 02.10.Yn, 02.30.Yy, 02.40.Pc, 02.40.Re, 02.50.Cw, 02.50.Sk, 07.05.Mh, 89.20.Ff, 89.75.-k, 89.75.Da, 89.75.Fb, 89.75.Hc, 89.75.Kd, 20N25, 28E10, 93B07, 93B51

## Abstract

Network science-based analysis of the observability of dynamical systems has been a focus of attention over the past five years. The maximum matching-based approach provides a simple tool to determine the minimum number of sensors and their positions. However, the resulting proportion of sensors is particularly small when compared to the size of the system, and, although structural observability is ensured, the system demands additional sensors to provide the small relative order needed for fast and robust process monitoring and control. In this paper, two clustering and simulated annealing-based methodologies are proposed to assign additional sensors to the dynamical systems. The proposed methodologies simplify the observation of the system and decrease its relative order. The usefulness of the proposed method is justified in a sensor-placement problem of a heat exchanger network. The results show that the relative order of the observability is decreased significantly by an increase in the number of additional sensors.

## 1. Introduction

The placement of sensors significantly affects the performance of identification, state estimation as well as fault detection and isolation (FDI) algorithms. The goal-oriented placement of sensors for dynamical systems is a challenging task [1]. Parameter estimation-oriented information entropy-based optimal sensor placement was investigated in [2]. Based on robust information entropy, a Bayesian sequential sensor placement algorithm for multi-type of sensor is also proposed [3]. Computational Fluid Dynamics (CFD) models were generated to predict wind-flow that used the data of a sensor placement utilised with prediction-value joint entropy [4]. For fault detection and isolation, an incremental analytical redundancy relation (ARR)-based algorithm was introduced in [5]. Structural analysis-based sensor placement strategies place additional sensors to isolate otherwise undisguisable faults [6]. In the initial sensor-constrained problems, the number of sensors is given and the detection time has to be minimized [7], while, as far as time-constrained problems are concerned, the aim is to minimize the number of required sensors that ensure a predefined detection time. In the case of dynamical systems, the previously mentioned detection time is related to the relative degree of the system [8]. Besides good FDI performance, the optimal placement of the sensors should also ensure the observability of the systems [9].

Network representation-based structural controllability and observability analysis of dynamical systems is a hot topic in literature [10,11,12,13,14,15,16] since the introduction of this new approach [17]. The methodology utilizes the maximum matching algorithm to determine the minimum set of actuators and sensors required to grant structural controllability and observability for an arbitrary dynamical system. Although the results are very promising, the methodology can overestimate the number of necessary actuators [18] when the details of the edge dynamic are neglected [14].

When the observability analysis of the system is performed on a properly defined network, the problem concerning the observability analysis is reduced to a reachability problem [19]. In some cascaded systems, this highlights that the number of necessary sensors is significantly smaller than the size of the state variables, which makes the observation vulnerable and the relative degree, i.e., the minimum number of derivatives that is necessary to observe at least one signal of inputs, is very high. To deal with the vulnerability, Liu et al. proposed a methodology to grant robustness in the undirected representation of the system [20].

There is an almost infinite number of objectives that can be used to define a sensor placement problem [21]. In most of the cases, the optimization problem is formulated as that the instrumentation cost should be minimized and the precision of reconciled values should be ensured in a reliable, resilient and robust manner [22]. As the problem is NP-hard (non-deterministic polynomial-time hard), the mixed-integer programming-based approach is suitable to small-to-medium sized networks [23]. To handle the complexity of the problem, a heuristic approach is mostly followed. For example, to reconstruct the internal states of biochemical reaction systems with the minimum number of sensors, a graphical method has been developed [24]. Among population-based optimization algorithms, genetic algorithms (GAs) are the most frequently applied, e.g., GA was utilized to find a sensor configuration that minimizes cost and maximizes the reliability and observability of the system [25]. The main benefit of these gradient-free heuristic optimization methods is that they can be utilized with a wide range of models [26]. Simulated annealing (SA) is also widely employed optimization algorithm in sensor placement [27]. SA lends itself to be combined with other search algorithms, e.g., with local search heuristics [28] or other SA inner loop, which results in dual representation simulated annealing (DRSA) [29]. These techniques are often combined also in sensor placement, e.g., GA and SA were successfully integrated [30]. Recently, Particle Swarm Optimization (PSO) was applied in an optimal sensor location problem (OSLP) [31] and in the design of an ultrasonic Structural Health Monitoring (SHM) system [32]. According to these publications, heuristic optimization techniques are efficient in finding a small number of sensors with less time-consuming computations [33].

In this paper, to ensure observability and minimize the relative order of dynamical systems, two methodologies for sensor placement are introduced. The presented methods combine the metaheuristic SA optimization method with clustering. SA is chosen as it exhibits excellent levels of performance with regard to graph-represented combinatorial optimization (CO). The use of simulated annealing is strengthened by the fact that the relative degree of the system can be interpreted as a path in a network, and simulated annealing models a random walk on the search graph. Based on this concept, SA was applied to place micro-hydropowers in a water supply network [34] and network alignment in biological systems [35].

As sensors practically group the observed state variables, sensor placement can be considered as a special *k*-medoid clustering problem. This concept has been utilized in Ref. [36], where fuzzy c-means clustering algorithm was utilized to characterize the spatial distribution of the sensor placement problem.

The Clustering Large Applications (CLASA) algorithm [37] developed as a fast and robust solution to the well-known *k*-medoid clustering problem. The fundamental idea of the paper is that instead of considering the distance between the cluster centers and the clustered objects, a goal-oriented sensor-placement algorithm can be derived by the introduction of a problem-relevant objective function into the scheme of the CLASA algorithm. The second novelty of the paper is that the random search of the resulted algorithm is fine-tuned by calculating the selection probabilities based on network distance-related fuzzy membership functions.

The structure of the paper is the following. In Section 2.1, the theoretical foundations and nomenclature of the relative degree of multiple-input and multiple-output (MIMO) dynamical systems are introduced. Section 2.2 presents the maximum matching-based sensor placement algorithm that ensures structural observability. In Section 2.3, the proposed optimization algorithms are introduced. Section 3 provides examples of application. Finally, Section 4 concludes the paper.

## 2. Sensor Placement to Ensure Observability and Minimal Relative Order

In Section 2.1, the relative degree of MIMO systems is defined and the sensor placement task is considered to be an optimization problem. Section 2.2 presents the first step of our methodology, which places the sensors as the structural observability of the system is guaranteed. Section 2.3 presents the proposed simulated annealing-based clustering algorithms that optimally place the additional sensors to minimize the relative degree of the system.

### 2.1. Problem Formulation

A nonlinear MIMO system can be represented by state-space model:

(1) $$\begin{array}{cc} \dot{x}\left(t\right) & =f(x(t))+g(x(t))u(t), \end{array}$$

(2) $$\begin{array}{cc} y(t) & =h(x(t)), \end{array}$$

where the vectors x, u and y stand for the state variables, inputs and outputs, respectively.

The model can be linearized and the resultant linear approximation represented by a linear state-space model [38]:

(3) $$\begin{array}{cc} \dot{x}\left(t\right) & =Ax(t)+Bu(t), \end{array}$$

(4) $$\begin{array}{cc} y(t) & =Cx(t). \end{array}$$

When the number of state variables is denoted by *N*, the number of actuators by *M* and the number of sensors by *K*, then the matrices $A\in {ℜ}^{N\times N}$, $B\in {ℜ}^{N\times M}$ and $C\in {ℜ}^{K\times N}$ define how state variables influence each other, how the actuators influence the state variables, and how the sensors record the state variables, respectively.

For each pair of output $y_{i}$ and input $u_{j}$ (i=1,…,K and j=1,…,M), the relative degree of the system $r_{i,j}$ can be defined as the minimum number of derivatives of $y_{i}\left(t\right)$ that is directly influenced by the change in $u_{j}\left(t\right)$. The relative degree for an arbitrary output *i* is defined as $r_{i}=\min_{j=1}^{M}r_{i,j}$, which requires the observer to observe the effect of at least one of the inputs.

When the aim of the observer is to estimate the effect of the $d\left(t\right)\in {ℜ}^{N}$ disturbance vector that independently influences all state variables,

(5) $$\begin{array}{cc} \dot{x}\left(t\right) & =Ax(t)+Ed(t), \end{array}$$

(6) $$\begin{array}{cc} y_{i}\left(t\right) & =Cx(t), \end{array}$$

then E is an identity matrix $I_{N}$ and the relative order of the system is $r=max_{i=1}^{K}r_{i}$.

**Figure 1:**
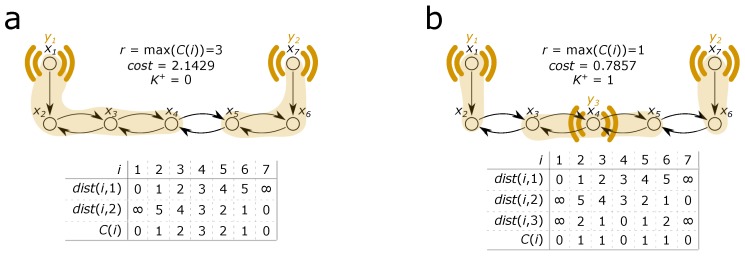
Illustration of the concept of relative degrees of the state variables and how one additional sensor decreases the relative degree of the system. (**a**) relative degrees in the case of two sensors that are necessary to grant observability; (**b**) relative degrees in the case of one additional sensor.

As Figure 1 illustrates the relative degree of an $x_{j}$ state variable can be interpreted as the dist(i,j) length of the path between the *j*-th state variable and the nearest sensor, C(i), selected as $\arg\min_{j}dist\left(i,j\right)$, while the relative order of the whole system is defined by the maximum of these minimal distances.

As the generation of a balanced placement of sensors is of interest, the set of sensor nodes *S* is determined by minimizing the following cost function:

(7) $$\min_{S}cost\left(G,S,\beta \right)=\beta \max_{i=1}^{K}r_{i}+\left(1-\beta \right)\frac{\sum_{i=1}^{K}r_{i}}{N},$$

where parameter β=[0,1] weights between the maximum and the balance-related average of the relative order of the system.

### 2.2. Placing Sensors to Ensure Observability

Besides the minimisation of the relative order, the observability of the system should also be ensured. Based on the Kalman rank criterion, a linear dynamical system is said to be structurally observable, if and only if the observability matrix $O={\left[{\left[C\right]}^{T},{\left[\mathrm{CA}\right]}^{T},{\left[{\mathrm{CA}}^{2}\right]}^{T},\ldots ,{\left[{\mathrm{CA}}^{N-1}\right]}^{T}\right]}^{T}$ is of full rank (rank(O)=N) [39].

**Figure 2:**
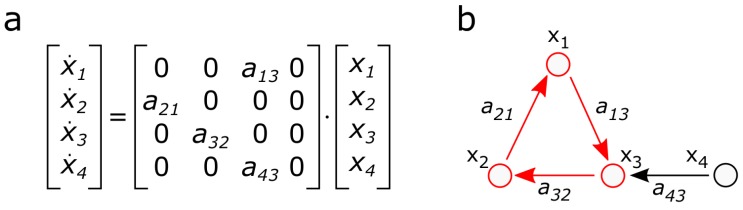
(**a**) example system x(t)=Ax(t); (**b**) graph representation of (**a**); state variables $\left\{x_{1},x_{2},x_{3}\right\}$ are matched by $MM=\left\{a_{13},a_{21},a_{32}\right\}=\left\{\left(x_{1},x_{3}\right),\left(x_{2},x_{1}\right),\left(x_{3},x_{2}\right)\right\}$ and the system is observable, since a sensor on $x_{4}$ can observe all state variables. If $x_{4}$ is removed from the system, then $\bar{V(MM)}$ is empty, which would mean that no sensor node could be identified as belonging to the system. This lack of the maximum matching-based approach can be eliminated by the path-finding method [14].

Based on the state-transition matrix A, a graph G(V,E) can be constructed where the set of vertices *V* represent the state variables and the edges E=V×V are determined by the nonzero elements of A (see Figure 2). In the graph representation, the relative degree $r_{i,j}$ can be defined as the distance between the input/disturbance $d_{j}$ and the output $y_{i}$, which is the distance between the *j*-th and *i*-th nodes in the directed network G(V,E), so the the optimization problem can be considered as a special graph partitioning problem. This graph-based representation is beneficial as the maximum matching of MM⊆E can be used to determine the minimal number and places of the sensor nodes that are required to ensure the observability of the system.

As directed graphs can be represented as bipartite graphs, the maximum matching of the related bipartite graph was studied [14,17]. The endpoints of the directed edges in MM are the matched nodes (V(MM)), while others are the unmatched nodes ($\bar{V(MM)}$), thus $V\left(MM\right)\cup \bar{V(MM)}=V$. Based on [17], the unmatched nodes $\bar{V(MM)}$ of G(V,E), where *E* is determined by $A^{T}$, define the driver nodes, i.e., where the actuators should be placed. Furthermore, unmatched nodes of G(V,E), where *E* is defined by A, should be the sensor nodes. In other words, the nodes where sensors should be placed. If the determined driver nodes or sensor nodes are assigned to the system, then it becomes a structurally controllable or structurally observable system [14]. As the aim of this paper is to deal with system observability, G(V,E) was used with the adjacency matrix based on A. To ensure the structural observability of the system, the path-finding method was used to define the minimum number of mandatory sensors and their location [14].

In order to generate the output matrix C such that the relative order is minimal and the system observable, first the unmatched set of nodes $S_{f}$ is generated, followed by the set of candidate sensor nodes $S_{c}$. The cardinality of the candidate sensor nodes is $K^{+}$ ($K^{+}=\left|S_{c}\right|$). The resulting C is designed based on the set of sensor nodes $S=S_{f}\cup S_{c}$ such that for each $x_{i}\in S$, $c_{j}$ is a one-hot row vector whose *i*-th element is non-zero, so $C={\left[c_{1}^{T},\ldots ,c_{K}^{T}\right]}^{T}$.

As the number of sensors which can be allocated without any restrictions is $K^{+}$, $\frac{K^{+}!}{|V\backslash S_{f}|!(|V\backslash S_{f}\left|-K^{+}!\right)}$ different combinations of possible placements of sensors exists. To solve this NP-hard problem, simulated annealing-based heuristic optimization algorithms are proposed in the following section.

### 2.3. Simulated Annealing and Fuzzy Clustering-based Output Configuration Design

To optimize the relative order and smoothness of these orders, the objective function (Equation (7)), which can be considered as a linear combination of the maximum and average distance of the sensors from the observed state variables was defined. Based on this interpretation, the minimisation problem can also be seen as a *k*-medoid clustering problem, where the centroid elements are the sensor nodes and the members of the clusters are the observed state variables.

To determine the locations of the additional sensors two approaches are proposed inspired by Clustering Large Applications based on Simulated Annealing algorithm (CLASA) [37] and the Geodesic Distance-based Fuzzy *c*-Medoid Clustering method (GDFCM) [40]. In the first algorithm, the CLASA algorithm is modified as follows. The original objective function of CLASA that calculates the distances between the objects and the medoids (in our case, the distances between the state variables and the sensors) is replaced by what was proposed in Equation (7). With the new objective function, not just the minimum relative order can be granted, but different sensor configurations with the same relative order can be distinguished to balance the load of the sensors. The search mechanism should be fine-tuned as the medoids of the fixed sensors ($S_{f}$) have fixed positions to ensure the observability of the system. The second algorithm enhances the random search of the resulted mCLASA algorithm by the introduction of distance-dependent selection probability calculated based on the Geodesic Distance-based Fuzzy *c*-Medoid Clustering method (GDFCM) [40].

In both algorithms, firstly the $S_{f}$ fixed sensor nodes are determined to grant the observability property based on the unmatched set of nodes. Then, the candidate sensor nodes $S_{c}$ are generated randomly, and the cost is calculated. Following this, in each iteration, one of the candidate sensor nodes ($s^{-}$) is randomly changed, and the new cost is calculated. From this, the difference between the new cost and the previously applied cost is calculated (Δ). The new placement of the sensors is accepted if Δ<0.

If Δ≥0, the worsened placement of the sensor is accepted only with the probability defined as $\exp(\frac{-\Delta }{T^{i}})$, where $T^{i}$ denotes the temperature of the cooling process of the SA in iteration *i*, whose dynamics can be seen in Equation (8). This randomization of the search allows for the algorithm to explore the search space and converge with the increase of the number of iterations:

(8) $$\;T^{i+1}=\alpha T^{i},\;\alpha ={\left(\frac{T_{min}}{T_{max}}\right)}^{1/maxiter}\;.$$

The pseudo code of the proposed modified Clustering Large Applications based on Simulated Annealing algorithm (mCLASA) can be seen in Algorithm 1. The algorithm possesses the following inputs and parameters: *G* denotes the network representation of the system from Equation (5), β∈(0,1) stands for the parameter of the cost function from Equation (7), $K^{+}$ represents the number of additional sensors, maxiter is the number of iterations of the simulated annealing, $T_{max}$ denotes the maximum, i.e., the initial temperature, and $T_{min}$ stands for the minimum temperature. The following values were used for the parameters: β=0.5, maxiter=500, $T_{max}=10$ and $T_{min}=10^{-2}$.

**Algorithm 1** Pseudo code of the modified CLASA algorithm (mCLASA).
1:**procedure**mCLASA(*G*, β, $K^{+}$, maxiter, $T_{max}$, $T_{min}$)2:    $\alpha ={\left(\frac{T_{min}}{T_{max}}\right)}^{1/maxiter}$, $T^{1}=T_{max}$3:    $S_{f}=getSensors\left(G\right)$              ▷ using the path finding method: [14]4:    Let $S_{c}$ be the set of the $K^{+}$ randomly chosen elements from $V\backslash S_{f}$5:    $cost=cost(G,S_{f}\cup S_{c},\beta )$                       ▷Equation (7)6:    **for** i=1 to maxiter **do**7:        Let $s^{-}$ be a randomly selected element from $S_{c}$8:        $S_{c}^{\prime }=S_{c}\backslash \left\{s^{-}\right\}$9:        Let $s^{+}$ be a randomly chosen node from $V\backslash (S_{f}\cup S_{c}^{\prime })$10:        $S_{c}^{\prime }=S_{c}^{\prime }\cup \left\{s^{+}\right\}$11:        $newcost=cost(G,S_{f}\cup S_{c}^{\prime },\beta )$12:        Δ=newcost−cost13:        **if** Δ<0 **then**14:           cost=newcost15:           $S_{c}=S_{c}^{\prime }$16:        **else**17:           **if** $random\left(\right)<\exp\left(\frac{-\Delta }{T^{i}}\right)$ **then**18:               cost=newcost19:               $S_{c}=S_{c}^{\prime }$20:           **end if**21:        **end if**22:        $T^{i+1}=\alpha T^{i}$23:    **end for**24:    **return** cost25:
**end procedure**



The second method utilizes the GDFCM method [40] to extend the previously introduced mCLASA method. This extension increases the probability that in each iteration potentially better neighboring sensors are selected to replace the sensors placed in the previous iteration steps $s^{-}$. The algorithm considers the sensors as cluster centers (medoids), determines the cluster assignments of the state variables, and swaps the medoid (the sensor) with a randomly selected state variable. The random selection utilizes distance-based fuzzy membership values. The cluster memberships $\mu_{i,j}$ of the state variables $R(s_{j})$ are determined by the fuzzy membership function (see Equation (9) in the description of the algorithm), where the distances are calculated in the undirected version of the graph ($G^{u}$). A roulette wheel selection [41] selects the new medoid according to the membership values. The search space of the algorithm is also controlled by the fuzzy exponent *m* which is decreased in each iteration by $\alpha_{m}$, in the same manner as α in Equation (8).

The pseudo code of the suggested Geodesic Distance-based Fuzzy *c*-medoid Clustering with Simulated Annealing algorithm (GDFCMSA) can be seen in Algorithm 2. The algorithm possesses the following inputs and parameters: *G* denotes the network representation of the system, β∈(0,1) stands for the balance parameter of the cost function, $K^{+}$ represents the number of additional sensors, maxiter is the number of iterations of the simulated annealing, $m_{max}$ denotes the maximum fuzzy component value and $m_{min}$ stands for the minimum fuzzy component value. The following values were used for the parameters: β=0.5, maxiter=500, $T_{max}=10$, $T_{min}=10^{-2}$, $m_{max}=10$ and $m_{min}=2$.

**Algorithm 2** Pseudo code of the Geodesic Distance-based Fuzzy *c*-Medoid Clustering with Simulated Annealing algorithm (GDFCMSA).
1:**procedure**GDFCMSA(*G*, β, $K^{+}$, maxiter, $T_{max}$, $T_{min}$, $m_{max}$, $m_{min}$)   2:    $\alpha ={\left(\frac{T_{min}}{T_{max}}\right)}^{1/maxiter}$, $T^{1}=T_{max}$, $\alpha_{m}={\left(\frac{m_{min}}{m_{max}}\right)}^{1/maxiter}$, $m^{1}=m_{max}$3:    $S_{f}=getSensors\left(G\right)$                ▷ using the path finding method: [14]  4:    Let $S_{c}$ be the set of the $K^{+}$ randomly chosen state elements from $V\backslash S_{f}$5:    $cost=cost(G,S_{f}\cup S_{c},\beta )$                        ▷ Equation (7)6:    **for** i=1 to maxiter **do**7:        Calculate the fuzzy membership values for $G^{u}$, 1≤i≤K and 1≤j≤N:
(9)$$\mu_{i,j}=\frac{1}{\sum_{k=1}^{K}{\left(\frac{r_{i,j}}{r_{k,j}}\right)}^{\frac{2}{m^{i}-1}}}$$8:        Let $s^{-}$ be a randomly selected sensor node from $S_{c}$9:        $S_{c}^{\prime }=S_{c}\backslash \left\{s^{-}\right\}$10:        $R\left(s^{-}\right)=\left\{x_{j}|x_{j}\in V,s^{-}=\overset{K}{\underset{k=1}{\arg\;\max\;}}\mu_{k,j}\right\}$11:        **for all** $s_{j}\in R\left(s^{-}\right)$ **do**12:           $P\left(s_{j}\right)=\mu_{s^{-},s_{j}}$13:        **end for**14:        $s^{+}=\left\{x_{j}|x_{j}=roulette\_wheel\_selection\left(P\right)\right\}$15:        $S_{c}^{\prime }=S_{c}^{\prime }\cup \left\{s^{+}\right\}$16:        $newcost=cost(G,S_{f}\cup S_{c}^{\prime },\beta )$17:        Δ=newcost−cost18:        **if** Δ<0 **then**19:           cost=newcost20:           $S_{c}=S_{c}^{\prime }$21:        **else**22:           **if** $random\left(\right)<\exp\left(\frac{-\Delta }{T^{i}}\right)$ **then**23:               cost=newcost24:               $S_{c}=S_{c}^{\prime }$25:           **end if**26:        **end if**27:        $T^{i+1}=\alpha T^{i}$, $m^{i+1}=\alpha_{m}m^{i}$28:    **end for**29:    **return** cost30:
**end procedure**



## 3. Results

In this section, four case studies will be introduced to demonstrate the applicability of the previously presented methods. Firstly, the details of the case studies will be given. We evaluate the proposed methods from two aspects. Firstly, the effect of the additional sensor nodes on the cost function and to the relative order of the system is studied; secondly, the speed of converge is analyzed.

### 3.1. Description of the Case Studies

**Figure 3:**
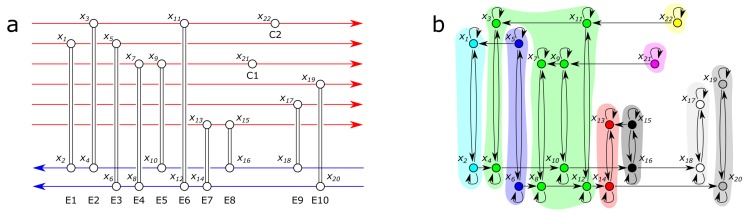
(**a**) the structure of the studied heat exchanger network represents how cold and hot streams influence each other; (**b**) the network representation of the state variables are related to the temperatures of the streams depicted in (**a**). Colors indicate strongly connected components.

The first case study is related the control of Heat Exchanger Networks (HENs). HENs are widely studied dynamical systems because the complexity of interlinked heat exchangers requires advanced process monitoring and control algorithms. The network topology of the studied HEN is shown in Figure 3. The network consists of six hot streams, two cold streams, ten heat exchanger cells and two utility coolers [42]. The state-transition matrix A of the problem can be easily determined based on the structure of the process [19], which results in results in 22 state variables.

Following the detailed analysis of the problem with regard to the placement of sensors in the HEN, three other dynamical systems will be analyzed to illustrate the applicability of the methods on larger examples. These benchmark examples are not typical control-relevant problems as they are used to study model-reduction algorithms [43], but these problems illustrate how the algorithms are able to detect structurally efficient placements. The first two additional examples are based on Modified Nodal Analysis (MNA) [44] and have 578 and 980 state variables, respectively. The last case study is based on the state space model of a partial element equivalent circuit (PEEC) of a patch antenna structure with 172 inductances, 6990 mutual inductances, and 2100 capacitances that define 480 state variables [45]. Further information about the case studies can be found in the Supplementary Material of the paper.

The parameters of the resulted case studies are summarized in Table 1. The network representations of the case studies and the resulted sensor placements are presented in the Supplementary Material of the paper.

As random search-based techniques were evaluated, each result was evaluated based on 100 independent runs of the algorithms. The parameters of the algorithms were the same in each scenario: β=0.5, $T_{max}=10$, $T_{min}=10^{-2}$, $m_{max}=10$, $m_{min}=2$. The reduction rates α and $\alpha_{m}$ were determined by Equation (8), as can be seen in Algorithms 1 and 2.

### 3.2. Effect of the Additional Nodes and the Robustness of the Optimization Algorithms

In all the examples, firstly the $S_{f}$ set of fixed sensors that are required to ensure the observability of the system is determined. The number of sensors added to improve the dynamical properties of the system is represented by the cardinality of $|K^{+}|$.

**Figure 4:**
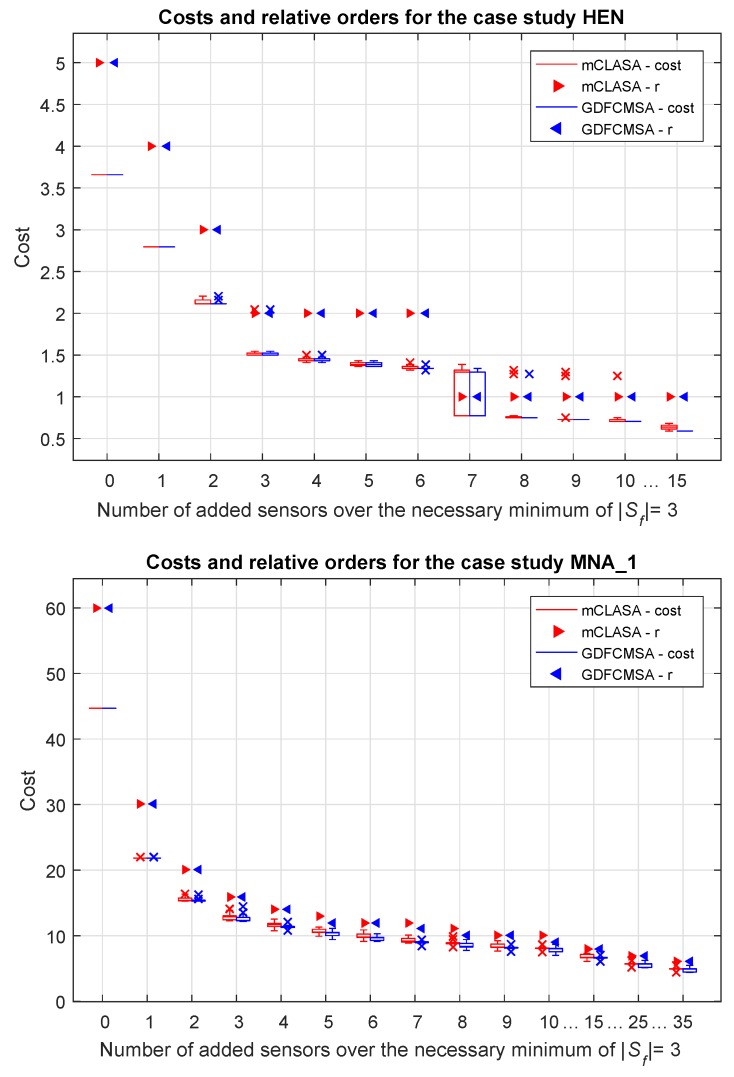
The values of the cost functions and relative degrees with regard to the number of added sensors in the case studies HEN and MNA_1. The box plots show the interquartile ranges and the medians of the cost functions, while the scatter plots visualize the minimum value of the relative order for 100 independent runs. For each value of the *x*-axis, the results provided by the mCLASA are shown on the left-hand side, and the results provided by the GDFCMSA algorithm are shown on the right-hand side. The triangles represent the relative orders, while the plus signs denote the outliers (if they exist) of the corresponding boxes.

**Figure 5:**
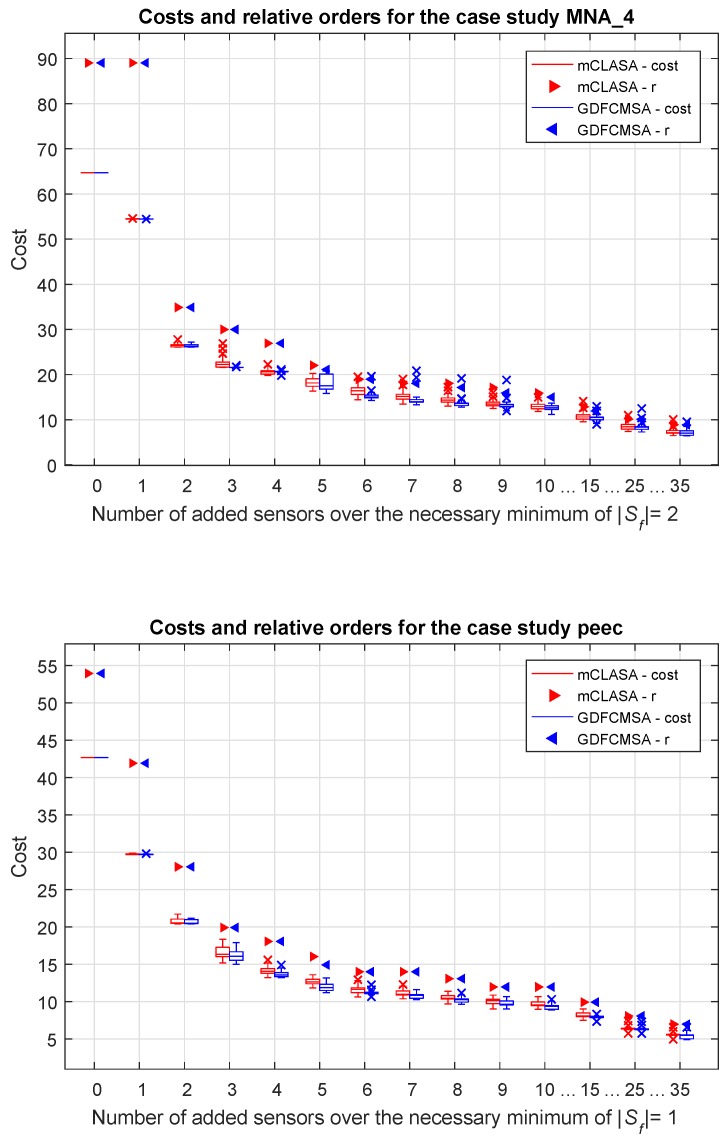
The values of the cost functions and relative degrees with regard to the number of added sensors in the case studies MNA_4 and peec. The box plots show the interquartile ranges and the medians of the cost functions, while the scatter plots visualize the minimum value of the relative order for 100 independent runs. For each value of the *x*-axis, the results provided by the mCLASA are presented on the left-hand side, while the results provided by the GDFCMSA algorithm are shown on the right-hand side. The triangles denote the relative orders, while the plus signs represent the outliers of the corresponding boxes.

To evaluate the robustness of the random search-based algorithms, the results of 100 independent runs were visualized in boxplots to show the distribution of the optimized cost functions. As can be seen in Figure 4 and Figure 5, the algorithms are consistent in most of the cases; however, the presence of outliers show that the algorithms can fail in local optimums.

It is visible that in all cases both the cost functions and the relative orders of the observed state variables decrease significantly as the number of sensors increases, so a relatively small quantity of additional sensors can cause a noticeable improvement in terms of the relative order compared to the observer designed only to ensure observability. It can also be pointed out that additional sensors ($K^{+}=$ 25 or 35) do not significantly reduce the order of the system.

The heights of the box plots show the interquartile ranges of the optimized cost functions. Higher box plots reflect higher uncertainty in the solutions which could indicate the increased difficulty of the sensor placement problem. For example, in Figure 4, for $K^{+}=7$, the boxplot shows a much wider distribution than for other $K^{+}$ that can be explained there is a transient in the structure of the solutions.

### 3.3. Convergence Analysis

**Figure 6:**
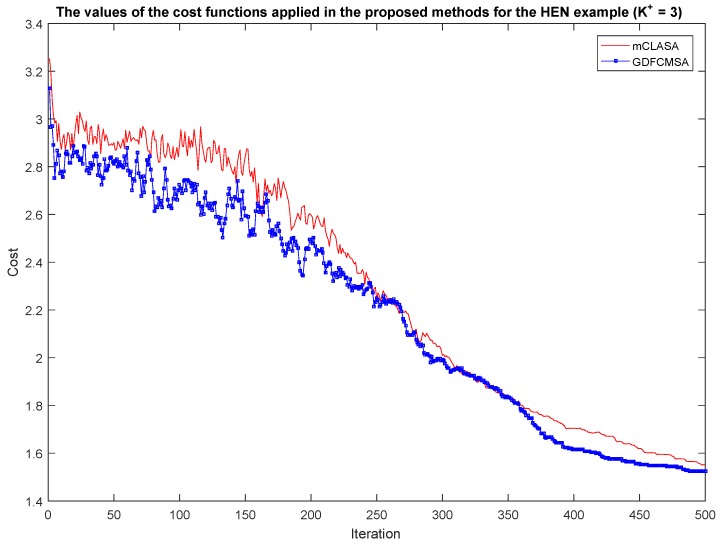
Analysis of the convergence with regard to the proposed methods in the case of the HEN. The colour red denotes the method mCLASA while the colour blue denotes the method GDFCMSA. It is clearly visible that the value of maxiter should be high because the methods also converge after 500 iterations. GDFCMSA provides a slightly better solution.

In the second phase of the analysis, the convergence of the proposed methods was tested. In Figure 6, the convergences of the methods can be seen for the HEN case study with the parameter setting $K^{+}=3$. The results and the network representation of the other case studies are presented in the Supplementary Material. In the figure, it can be seen that how fast the methods converge in terms of the number of iterations. With the addition of three sensors, the cost function was reduced by more than 50% over the 500 iterations, which illustrate well the benefit of the proposed methods.

In this figure, it can also be seen that it is worth selecting a high value of the parameter maxiter because, even during the 500th iteration, the cost functions still show a small decrease.

From this figure, it is evident that the fuzzy extension of the algorithm improves the speed of convergence which is also reflected from the slightly better performances shown in Figure 4 and Figure 5.

The durations of the calculations were also measured with maxiter=500 to provide an illustrative comparison between the algorithm and the complexity of the problem. The results are presented in Table 2. The durations of calculations are valid for our MATLAB implementation (R2016a, The MathWorks, Natick, MA, USA) when run on a notebook using Windows 10 (Microsoft, Redmond, WA, USA), an Intel Core i7-6600U processor (Santa Clara, CA, USA) and 16 GB of RAM.

The short running times shown in Table 2 raise the question of whether the exhaustive enumeration of the solutions could handle the problem and if the heuristic search is necessary. The problem is combinatorially complex, for each $K^{+}$ N−|Sf|K+ possible solutions should be examined. In the HEN case study for $K^{+}=9$, the SA examines only 500 solutions instead of the 92,378 possible configurations. For larger problems, the difference is more significant, e.g., the example of MNA_4 with $K^{+}=35$ would require 97835≈2.4×1064 function evaluations, although this problem cannot be considered as a huge system.

## 4. Conclusions

To ensure the structural observability in addition to fast and robust observer response, two clustering- and simulated annealing-based methods were proposed.

Additional sensors are placed into the system based on the CLASA algorithm by the mCLASA algorithm. The placements of additional sensors is further improved by the Geodesic Distance-based Fuzzy *c*-Medoid Clustering with Simulated Annealing algorithm (GDFCMSA) as their positioning is based on a geodesic distance-based fuzzy membership functions. Simulated annealing is applied by both algorithms to minimize the cost function, which in turn minimizes the maximum and average of the relative orders of the system to generate a balanced placement of sensors.

A slightly better solution is provided by the GDFCMSA algorithm than the mCLASA method at the expense of some additional computational resources for the evaluation of the membership degrees. Solutions to massive problems are generated by both methods over a short period of time, which illustrates the applicability of these methods in an industrial setting.

Although this paper deals with the placement of sensors, the proposed methodology can be applied to control configuration design as well, since this is the dual problem of the design of the output configuration.

## Figures and Tables

**Table 1:** The main parameters of the networks studied in the examples. |V| denotes the number of state variables, |E| stands for the number of the connections between the state variables, $|S_{f}|$ represents the number of fixed sensor nodes, and $K^{+}$ denotes the number of additional nodes to be analyzed.

Network	Description	|V|	|E|	$|S_{f}|$	$K^{+}$
HEN	A simple heat exchanger network.	22	56	3	0,…,15
MNA_1	Modified Nodal Analysis of a multiport, voltage sources.	578	1694	3	0,…,35
MNA_4	Modified Nodal Analysis of a multiport, voltage sources.	980	2872	2	0,…,35
peec	Partial element equivalent circuit (PEEC) model.	480	1346	1	0,…,35

**Table 2:** Durations of calculations in seconds in terms of the optimal placement of $K^{+}$ additional sensors. The presented results are the means of 100 independent runs.

$K^{+}$	HEN		MNA_1		MNA_4		peec	
	mCLASA	GDFCMSA	mCLASA	GDFCMSA	mCLASA	GDFCMSA	mCLASA	GDFCMSA
1	0.0158 s	0.0164 s	0.1311 s	0.1396 s	0.1795 s	0.1892 s	0.0704 s	0.0715 s
2	0.0173 s	0.0183 s	0.1752 s	0.1689 s	0.2240 s	0.2306 s	0.0945 s	0.0955 s
3	0.0205 s	0.0227 s	0.2230 s	0.2120 s	0.2676 s	0.2922 s	0.1176 s	0.1179 s
5	0.0233 s	0.0280 s	0.2638 s	0.2999 s	0.3724 s	0.3749 s	0.1602 s	0.1614 s
10	0.0310 s	0.0315 s	0.3965 s	0.4106 s	0.6353 s	0.7106 s	0.2829 s	0.2850 s
15	0.0427 s	0.0428 s	0.5984 s	0.6721 s	0.9049 s	0.9176 s	0.4631 s	0.4237 s
25	n.a.	n.a.	0.8052 s	0.8170 s	1.5417 s	1.4754 s	0.6385 s	0.6862 s
35	n.a.	n.a.	1.3258 s	1.3404 s	2.1008 s	2.0255 s	0.9229 s	1.0077 s

## Supplementary Materials

The supplementary material of the paper (additional figures, MATLAB source codes, the details of the networks) is available from https://www.abonyilab.com/network-science/controlability-and-observability.

## Abbreviations

Abbreviations used in this article:

CLASA:	Clustering Large Applications based on Simulated Annealing
GDFCM:	Geodesic Distance-based Fuzzy c-Medoid Clustering method
GDFCMSA:	Geodesic Distance-based Fuzzy c-Medoid Clustering with Simulated Annealing method
HEN:	Heat Exchanger Network
mCLASA:	modified Clustering Large Applications based on Simulated Annealing
MIMO:	Multiple-input and multiple-output
SA:	Simulated Annealing

## Nomenclature

x	vector of state variables
u	vector of inputs
d	vector of disturbances
y	vector of outputs
A	state-transition matrix
B	input matrix
E	disturbance matrix
C	output matrix
O	observability matrix
*N*	number of state variables
*M*	number of inputs
*K*	number of outputs
$K^{+}$	number of additional sensor nodes
$b^{j}$	*j*th column of matrix B
$b_{j}$	*j*th row of matrix B
$r_{i,j}$	relative degree of $y_{i}$ and $u_{j}$
$r_{i}$	relative degree of $y_{i}$
*r*	relative order of the system
G(V,E)	network representation of the system
*V*	set of vertices of the network representation of the system
*E*	connections between the state variables
$G^{u}$	undirected representation of *G*
*S*	set of sensor nodes
$S_{f}$	set of fixed sensor nodes
$S_{c}$	set of candidate sensor nodes
β	weighting parameter of cost function
MM	edges of a maximum matching
V(MM)	matched nodes
$\bar{V(MM)}$	unmatched nodes
Δ	difference between cost functions in SA
$T_{max},T_{min}$	maximum and minimum temperature in SA
$m_{max},m_{min}$	maximum and minimum fuzzy exponent in SA
$T^{i}$	temperature in iteration *i*
$m^{i}$	fuzzy exponent in iteration *i*
α	reduction rate of temperature
$\alpha_{m}$	reduction rate of the fuzzy exponent
maxiter	number of iterations of SA
$R(s_{j})$	set of the state variables of the cluster of sensor node $s_{j}$
$\mu_{i,j}$	fuzzy membership function
