# Supplementary material for: Network Distance-Based Simulated Annealing and Fuzzy Clustering for Sensor Placement Ensuring Observability and Minimal Relative Degree
Source: Sensors (Basel). 2018 Sep 14;18(9):3096. doi: 10.3390/s18093096 (PMC6164052; doi:10.3390/s18093096)
Supplement: Supplementary file 1 [file sensors-18-03096-s001.pdf]

# Supplementary material - Network distance-based simulated annealing and fuzzy clustering for sensor placement ensuring observability and minimal relative degree

Daniel Leitold[1,2], Agnes Vathy-Fogarassy[1,2] and Janos Abonyi[2]

[1]Department of Computer Science and Systems Technology,
University of Pannonia, Egyetem u. 10, H-8200 Veszprém, Hungary
[2]MTA-PE Lendület Complex Systems Monitoring Research Group
University of Pannonia, Egyetem u. 10., POB. 158, H-8200 Veszprém, Hungary

## I Introduction

The sensor placement methodology is presented through three different problems. The first one is a Heat Exchanger Network (HEN), the second one is the Modified Nodal Analysis (MNA), while the last one is Partial Element Equivalent Circuit (PEEC). The proposed methodology was motivated by the practically relevant HEN problem; therefore, the details of this case study are introduced in the paper. To present some characteristics of the method, we used two larger MNA problems and one larger PEEC problem. This supplementary material aims to make these problems more understandable by giving details about the structure of these problems and the placed networks.

The supplementary material is organized as follows. In Section II, we introduce the simple versions of the applied case studies. In Section III, we show the network-based presentation of the case studies with the fixed sensors ($S_f$) and the selected optimal candidate sensors ($S_c$), while in Section IV we present the convergence of the algorithms.

## II Details of the case studies

### II.1 The modified nodal analysis problem

In the MNA problem voltage sources are connected to the port of multiport to determine its the admittance matrix [2]. A simple two-port RLC circuit example can be seen in Figure S1.

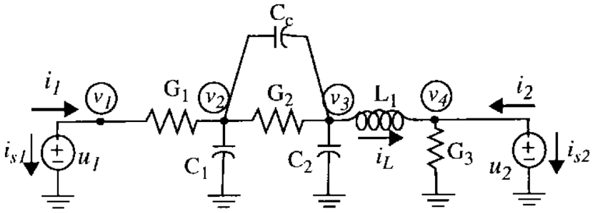


Figure S1: A simple two-port RLC circuit.

With the voltages ($\boldsymbol{u}$) and currents ($\boldsymbol{i}$) the multiport can be represented with the MNA equations, Eq. (1) and Eq. (2).

$$
\begin{aligned}
\mathbf{C}\dot{x} &= \mathbf{G}\boldsymbol{x} + \mathbf{B}\boldsymbol{u} \\
\boldsymbol{i} &= \mathbf{L}^{\mathbf{T}}\boldsymbol{x}
\end{aligned} \tag{1}
$$

$$
\begin{aligned}
\begin{bmatrix} 0 & 0 & 0 & 0 & 0 & 0 & 0 \\ 0 & (C_c + C_1) & -C_c & 0 & 0 & 0 & 0 \\ 0 & -C_c & (C_2 + C_c) & 0 & 0 & 0 & 0 \\ 0 & 0 & 0 & 0 & 0 & 0 & 0 \\ 0 & 0 & 0 & 0 & L_1 0 & 0 & 0 \\ 0 & 0 & 0 & 0 & 0 & 0 & 0 \\ 0 & 0 & 0 & 0 & 0 & 0 & 0 \end{bmatrix} \begin{bmatrix} \dot{v}_1 \\ \dot{v}_2 \\ \dot{v}_3 \\ \dot{v}_4 \\ \dot{i}_L \\ \dot{i}_{s1} \\ \dot{i}_{s2} \end{bmatrix} &= - \begin{bmatrix} G_1 & -G_1 & 0 & 0 & 0 & 1 & 0 \\ -G_1 & (G_1 + G_2) & -G_2 & 0 & 0 & 0 & 0 \\ 0 & -G_2 & G_2 & 0 & 1 & 0 & 0 \\ 0 & 0 & 0 & G_3 & -1 & 0 & 1 \\ 0 & 0 & -1 & 1 & 0 & 0 & 0 \\ -1 & 0 & 0 & 0 & 0 & 0 & 0 \\ 0 & 0 & 0 & -1 & 0 & 0 & 0 \end{bmatrix} \begin{bmatrix} v_1 \\ v_2 \\ v_3 \\ v_4 \\ i_L \\ i_{s1} \\ i_{s2} \end{bmatrix} + \begin{bmatrix} 0 & 0 \\ 0 & 0 \\ 0 & 0 \\ 0 & 0 \\ 0 & 0 \\ -1 & 0 \\ 0 & -1 \end{bmatrix} \begin{bmatrix} u_1 \\ u_2 \end{bmatrix} \\
\begin{bmatrix} i_1 \\ i_2 \end{bmatrix} &= \begin{bmatrix} 0 & 0 & 0 & 0 & 0 & -1 & 0 \\ 0 & 0 & 0 & 0 & 0 & 0 & -1 \end{bmatrix} \begin{bmatrix} v_1 \\ v_2 \\ v_3 \\ v_4 \\ i_L \\ i_{s1} \\ i_{s2} \end{bmatrix}
\end{aligned} \tag{2}
$$

The proposed method utilizes the network representation of this state transition matrix depicted in Figure S2.

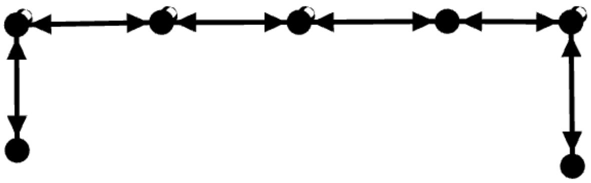

Figure S2: Network representation of the simple two-port RLC circuit.

This simple example demonstrated only the basic building blocks of the MNA problems. The structures of the studied more complex MNA problems will be presented in the following section (on Figure S6 and Figure S7).

## II.2 The partial element equivalent circuit problem

The smallest possible example for the PEEC problem can be seen in Figure S3.

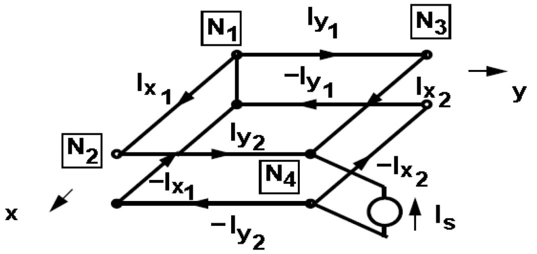


Figure S3: The smallest PEEC problem for two planes [3].

As can be seen, PEEC is used to model three-dimensional interconnects. Partial inductance included by each of the connection. If node $N_1$ is shorted and into node $N_4$ current is injected, then the self inductance of $N_4$ is $L_{44} = \frac{V_4}{sI}$ [1]. The circuit equations can be given as in Eq. (3).

$$
\begin{bmatrix} 0 & 0 & 0 & 0 & 1 & 0 & 1 & 0 & 1 \\ 0 & 0 & 0 & 0 & -1 & 0 & 0 & 1 & 0 \\ 0 & 0 & 0 & 0 & 0 & 1 & -1 & 0 & 0 \\ 0 & 0 & 0 & 0 & 0 & -1 & 0 & -1 & 0 \\ 1 & -1 & 0 & 0 & -Lx_{11} & -Lx_{12} & 0 & 0 & 0 \\ 0 & 0 & 1 & -1 & -Lx_{12} & -Lx_{22} & 0 & 0 & 0 \\ 1 & 0 & -1 & 0 & 0 & 0 & -Ly_{11} & -Ly_{12} & 0 \\ 0 & 1 & 0 & -1 & 0 & 0 & -Ly_{12} & -Ly_{22} & 0 \\ 1 & 0 & 0 & 0 & 0 & 0 & 0 & 0 & 0 \end{bmatrix} \begin{bmatrix} V_{N1} \\ V_{N2} \\ V_{N3} \\ V_{N4} \\ sI_{x1} \\ sI_{x2} \\ sI_{y1} \\ sI_{y2} \\ sI_{sh} \end{bmatrix} = \begin{bmatrix} 0 \\ 0 \\ 0 \\ I_s \\ 0 \\ 0 \\ 0 \\ 0 \\ 0 \end{bmatrix} \tag{3}
$$

The network representation of the matrix of of state variables is depicted in Figure S4.

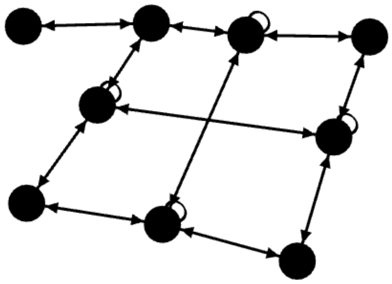

Figure S4: Network representation of the example problem for two planes.

This simple example demonstrated only the basic building blocks of the PEEC problem. The structure of the studied more complex problem will be presented in the following section (on Figure S8).

## III Network representations of the case studies

The network representations contain the fixed ($S_f$) and candidate ($S_c$) sensors noted with turquoise and green, respectively. As the results of the mCLASA and GDFCMSA methods can differ, both results are presented. The only exception is the HEN case study, as the two methods provide identical solutions. In the following, Figure S5, S6, S7 and S8 show the solutions provided by the two methods for the HEN, MNA_1, MNA_4 and peec case studies.

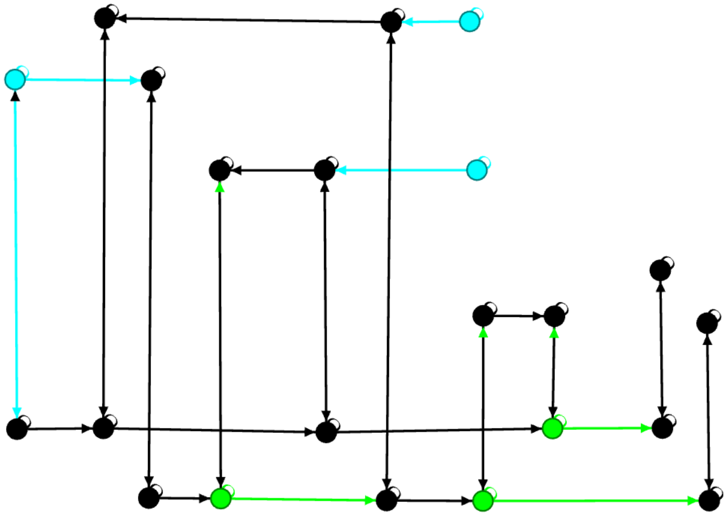

Figure S5: Network representation of the HEN case study at $K^+ = 3$ for both the mCLASA and GDFCMSA method. The turquoise dots denote the fixed sensors ($S_f$) while the green dots denote the placed additional sensors ($S_c$) determined by mCLASA.

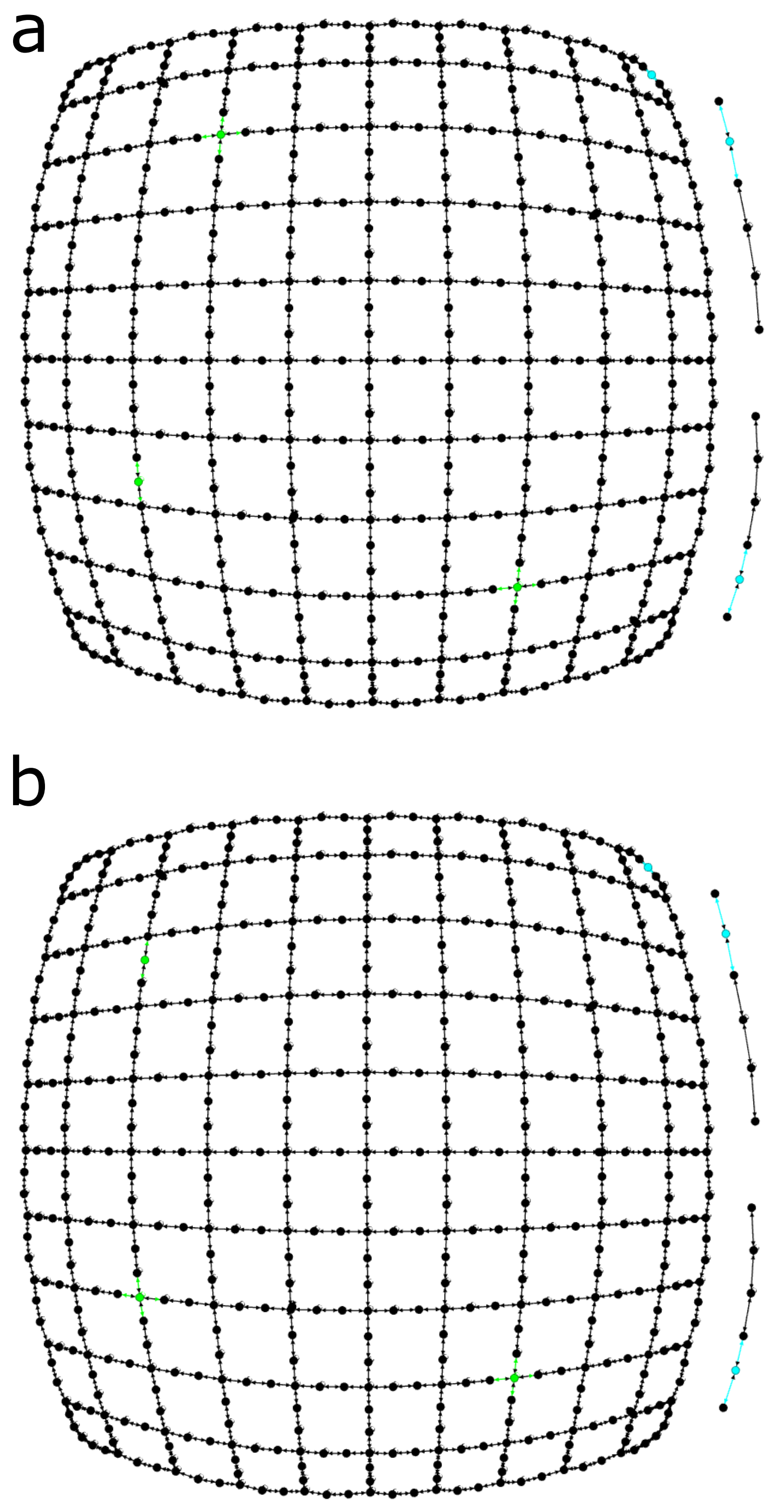


Figure S6: Network representation of the the MNA_1 case study at $K^+ = 3$ obtained by (**a**) the mCLASA and (**b**) the GDFCMSA methods. The turquoise dots denote the fixed sensors ($S_f$) while the green dots denote the optimal candidate sensors ($S_c$) determined by mCLASA.

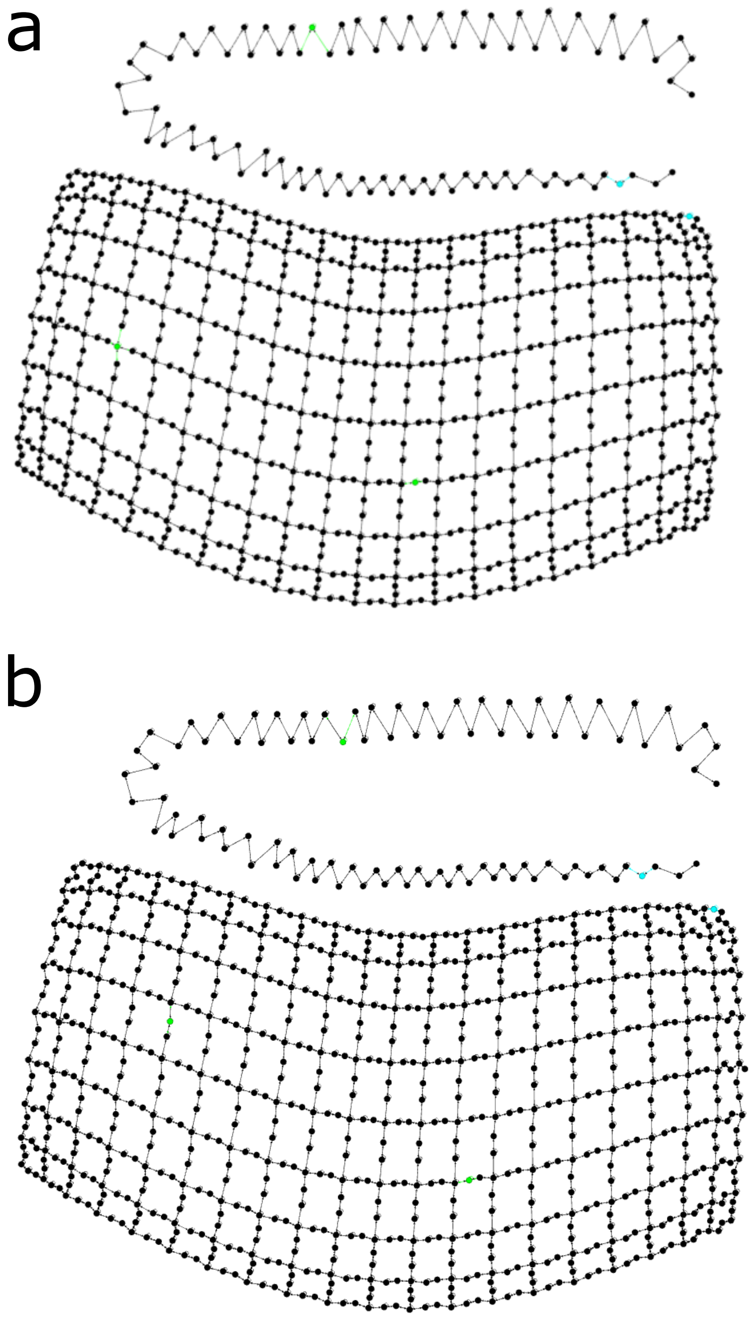


Figure S7: Network representation of the MNA_4 case study at $K^+ = 3$ obtained by (**a**) the mCLASA and (**b**) the GDFCMSA methods. The turquoise dots denote the fixed sensors ($S_f$) while green dots denote the optimal candidate sensors ($S_c$) determined by mCLASA.

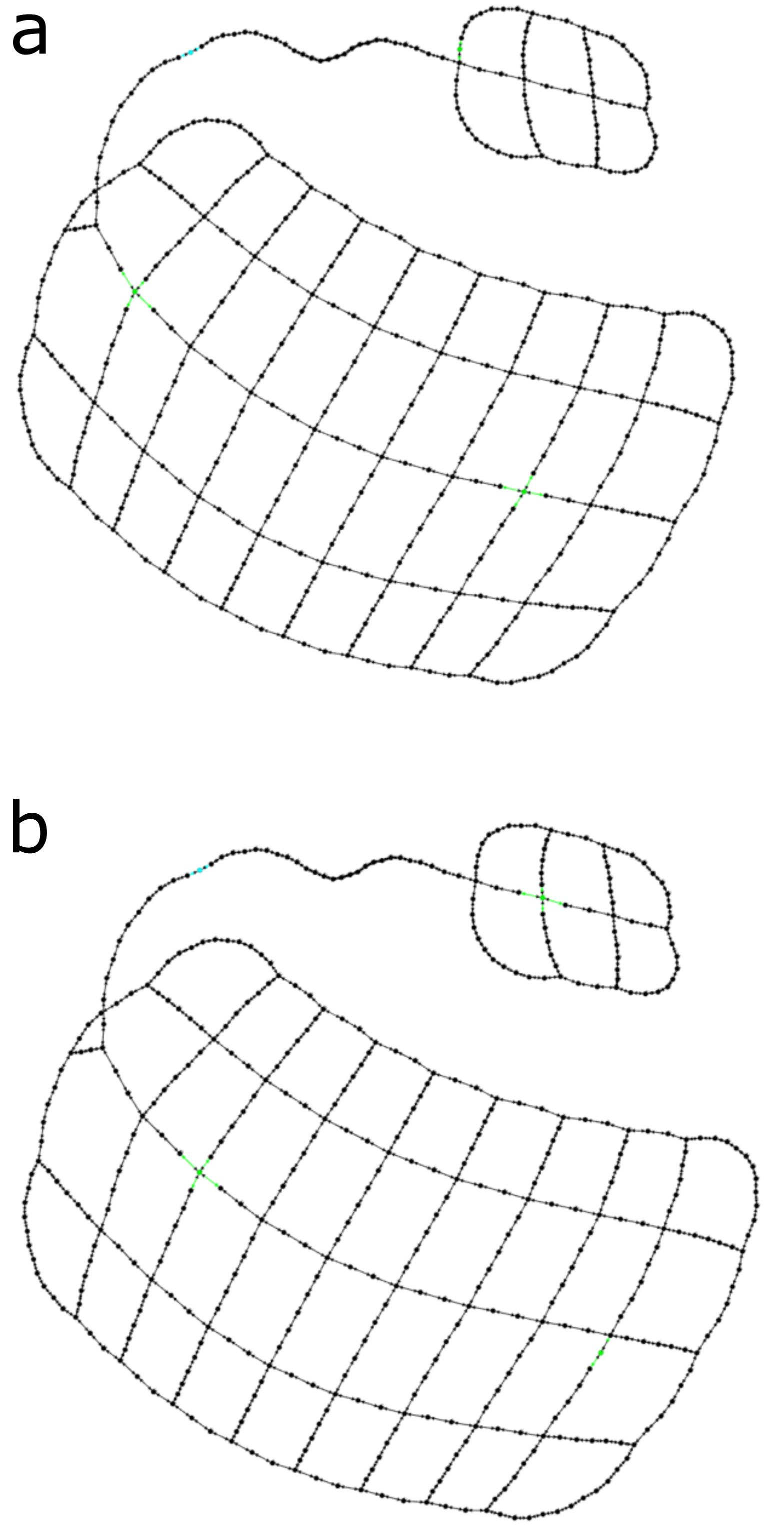


Figure S8: Network representation of the of the peec case study at $K^+ = 3$ obtained by (**a**) the mCLASA and (**b**) the GDFCMSA methods. The turquoise dots denote the fixed sensors ($S_f$) while green dots denote the optimal candidate sensors ($S_c$) determined by mCLASA.

# IV Convergence of the solutions

The converge of the HEN case study was introduced in the article. Here, the converge of the remaining three case studies are presented in Figures S9, S10, S11 and S12.

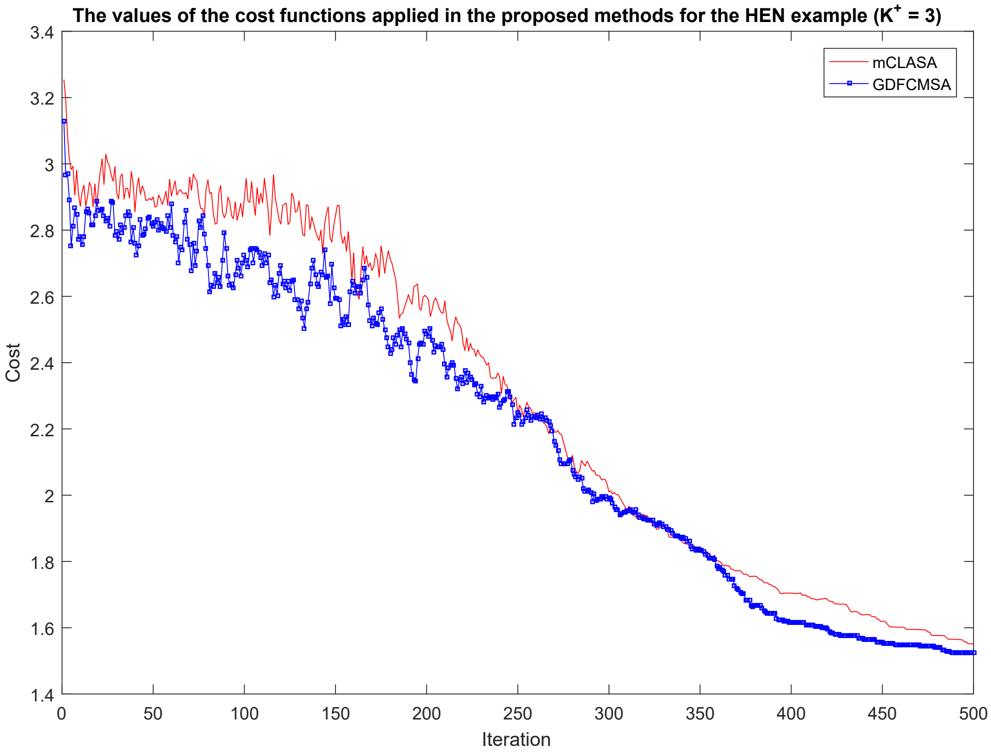


Figure S9: Convergence of the proposed methods in the case of case study HEN. The colour red denotes the method mCLASA while the colour blue denotes the method GDFCMSA.

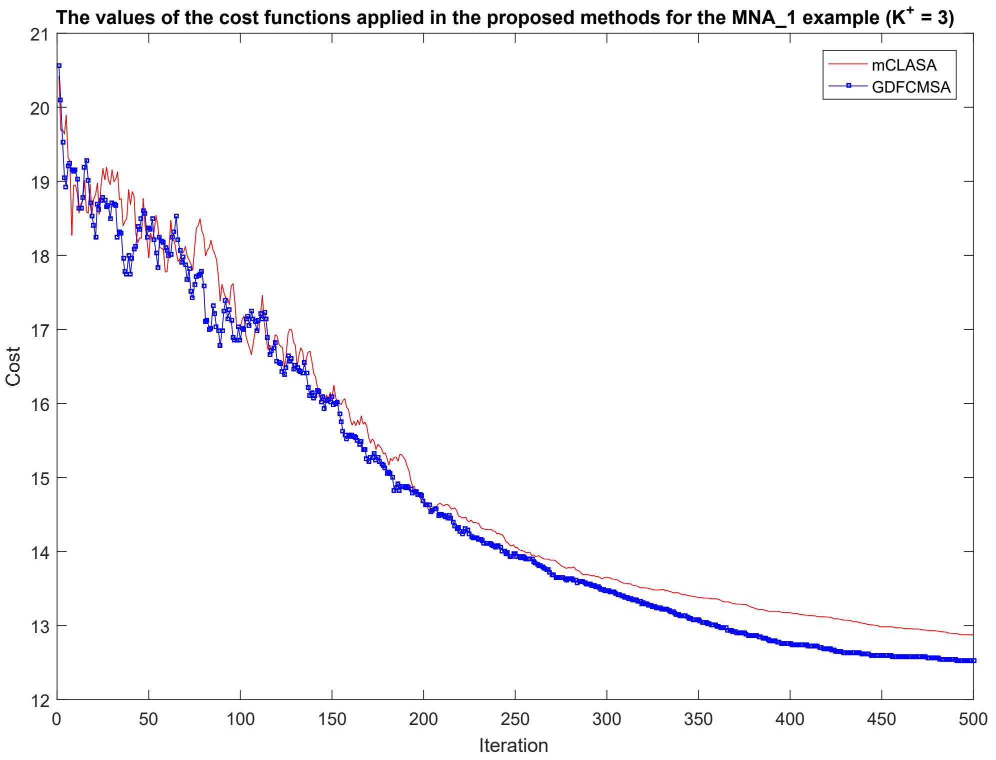


Figure S10: Convergence of the proposed methods in the case of case study MNA_1. The colour red denotes the method mCLASA while the colour blue denotes the method GDFCMSA.

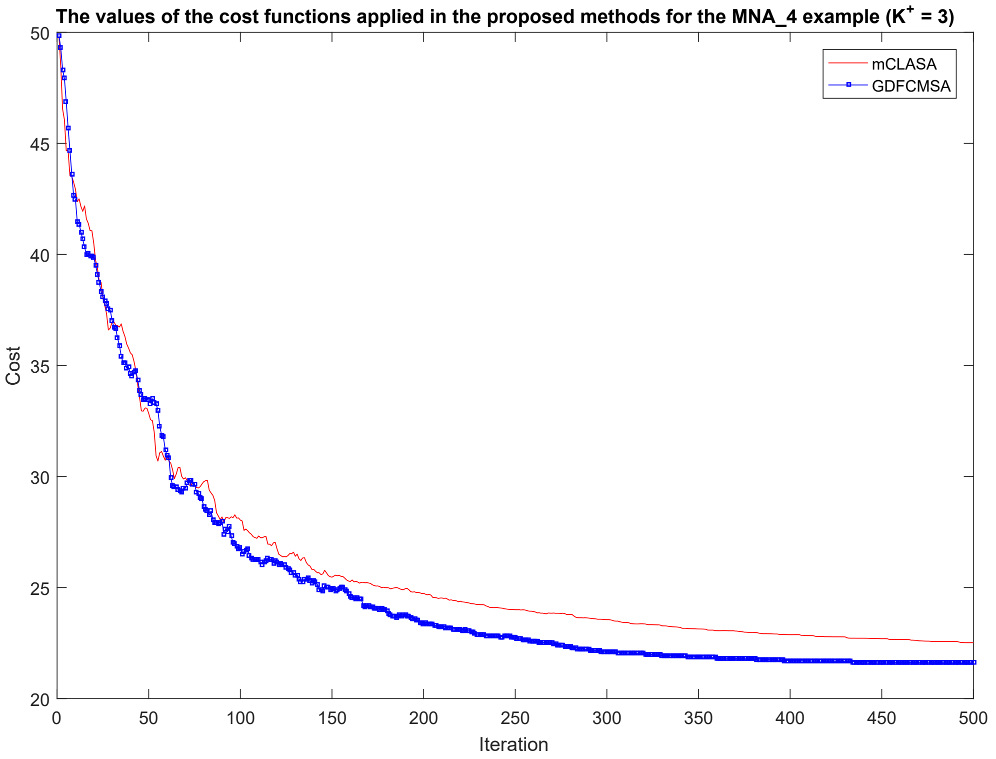


Figure S11: Convergence of the proposed methods in the case of case study MNA_4. The colour red denotes the method mCLASA while the colour blue denotes the method GDFCMSA.

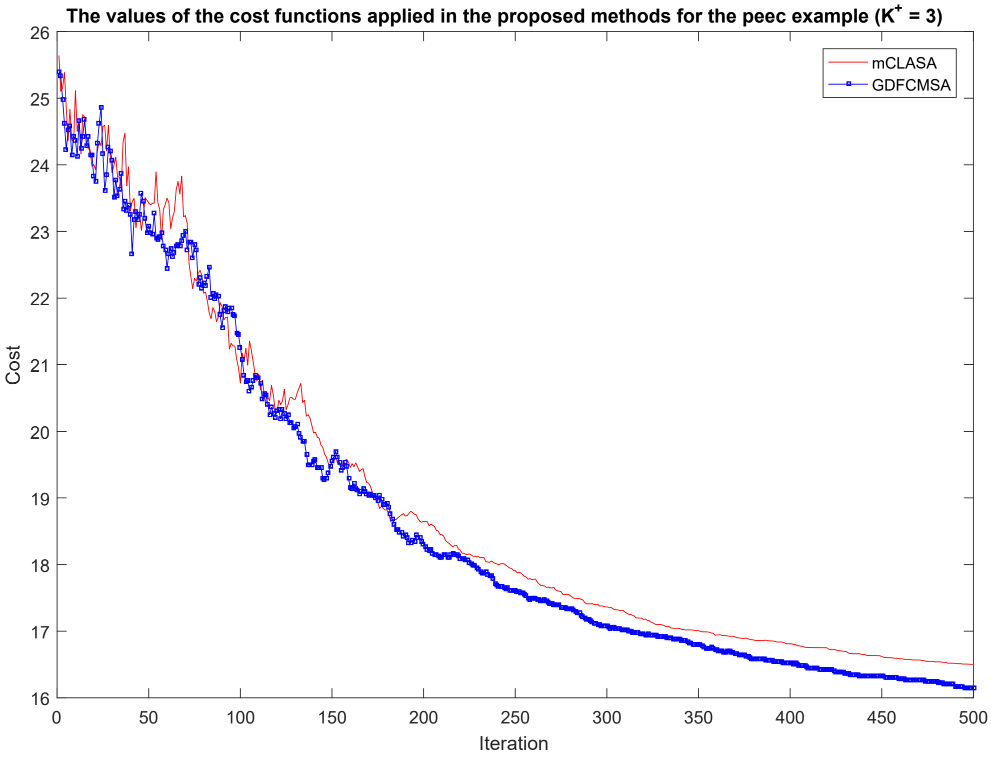


Figure S12: Convergence of the proposed methods in the case of case study peec. The colour red denotes the method mCLASA while the colour blue denotes the method GDFCMSA.

# References


[1] Hansruedi Heeb, Albert E Ruehli, J Eric Bracken, and Ronald A Rohrer. Three dimensional circuit oriented electromagnetic modeling for vlsi interconnects. In *Computer Design: VLSI in Computers and Processors, 1992. ICCD'92. Proceedings, IEEE 1992 International Conference on*, pages 218–221. IEEE, 1992.

[2] Altan Odabasioglu, Mustafa Celik, and Lawrence T Pileggi. Prima: Passive reduced-order interconnect macromodeling algorithm. In *Proceedings of the 1997 IEEE/ACM international conference on Computer-aided design*, pages 58–65. IEEE Computer Society, 1997.

[3] Fan Zhou, Albert E Ruehli, and Jun Fan. Efficient mid-frequency plane inductance computation. In *Electromagnetic Compatibility (EMC), 2010 IEEE International Symposium on*, pages 831–836. IEEE, 2010.
